# A Espessura que Engana: O Papel da Imagem, da Biópsia e da Genética na Avaliação da Hipertrofia Ventricular

**DOI:** 10.36660/abc.20260119

**Published:** 2026-04-16

**Authors:** Murillo de Oliveira Antunes

**Affiliations:** 1 Hospital das Clínicas da Faculdade de Medicina da Universidade de São Paulo Instituto do Coração São Paulo SP Brasil Instituto do Coração do Hospital das Clínicas da Faculdade de Medicina da Universidade de São Paulo, São Paulo, SP – Brasil; 2 Universidade São Francisco Bragança Paulista SP Brasil Universidade São Francisco (USF), Bragança Paulista, SP – Brasil

**Keywords:** Diagnóstico por Imagem, Biópsia, Genética, Cardiomiopatias

O aumento da espessura da parede ventricular é um achado comum na prática cardiovascular e é frequentemente atribuído à hipertrofia miocárdica secundária ou à cardiomiopatia hipertrófica (CMH) sarcomérica. No entanto, esse fenótipo pode mascarar um amplo espectro de doenças genéticas, infiltrativas e de armazenamento, cada uma associada a implicações prognósticas e estratégias terapêuticas distintas. A diferenciação precisa entre a verdadeira hipertrofia miocárdica e as fenocópias — ou genocópias — da CMH permanece um grande desafio diagnóstico e requer uma abordagem multimodal que integre exames de imagem cardíaca avançados, biópsia endomiocárdica (BEM) e testes genéticos.^1-3^

A imagem cardíaca evoluiu para além da avaliação morfológica. A ressonância magnética cardíaca (RMC) com caracterização tecidual se tornou um pilar da avaliação não invasiva em pacientes com aumento da espessura miocárdica. Técnicas de mapeamento paramétrico, incluindo mapeamento de T1 e T2 e quantificação do volume extracelular, permitem a detecção de alterações na composição miocárdica, como edema, fibrose e infiltração, fornecendo pistas diagnósticas importantes para condições como amiloidose cardíaca, doença de Fabry e cardiomiopatias de armazenamento de glicogênio (por exemplo, doença relacionada ao PRKAG2).^4-6^ Além disso, padrões específicos de realce tardio de gadolínio podem reforçar a suspeita de etiologias não sarcoméricas.^3,7^

Apesar dos avanços substanciais em exames de imagem, a avaliação histopatológica obtida por meio de BEM permanece o padrão ouro para o diagnóstico em casos selecionados. A análise tecidual permite a identificação definitiva de depósitos anormais, como fibrilas amiloides, bem como de anormalidades ultraestruturais, incluindo vacúolos preenchidos com glicogênio na cardiomiopatia PRKAG2, que podem não ser distinguidos de forma confiável apenas por exames de imagem. A BEM é particularmente valiosa quando os achados de imagem são inconclusivos ou quando a confirmação de um diagnóstico específico é necessária para orientar terapias modificadoras da doença.^8,9^

Os testes genéticos transformaram o panorama diagnóstico das cardiomiopatias hereditárias, permitindo a confirmação da CMH sarcomérica e a identificação de suas fenocópias.^10-12^ No entanto, a avaliação genética introduz complexidade adicional, incluindo sobreposições fenotípicas e a detecção frequente de variantes em múltiplos genes. Nesses cenários, a BEM recupera importância crucial, uma vez que os achados histopatológicos podem auxiliar no estabelecimento da correlação genótipo-fenótipo e na identificação da variante causal, orientando, assim, o manejo clínico e as estratégias de rastreamento familiar.

Em conclusão, a avaliação do aumento da espessura da parede miocárdica exige uma abordagem diagnóstica integrada.^13^ A RMC desempenha um papel central na triagem e na caracterização tecidual; no entanto, a BEM está longe de ser obsoleta, fornecendo um diagnóstico definitivo em nível tecidual. Quando combinada com testes genéticos, a BEM aumenta a precisão diagnóstica, esclarece a etiologia subjacente da aparente "hipertrofia" e apoia uma abordagem de medicina de precisão no tratamento contemporâneo da cardiomiopatia.
