# Supplementary material for: A Espessura que Engana: O Papel da Imagem, da Biópsia e da Genética na Avaliação da Hipertrofia Ventricular
Source: Arq Bras Cardiol. 2026 Apr 16;123(2):e20260119. [Article in Portuguese] doi: 10.36660/abc.20260119 (PMC13128184; doi:10.36660/abc.20260119)
Supplement: Translation

# Translation: The Thickness That Deceives: The Role of Imaging, Biopsy, and Genetics in the Evaluation of Ventricular Hypertrophy

**DOI:** 10.36660/abc.20260119i

**Authors:** Murillo de Oliveira Antunes

**Affiliations:** 1 São Paulo SP Brazil Instituto do Coração do Hospital das Clínicas da Faculdade de Medicina da Universidade de São Paulo, São Paulo, SP – Brazil; 2 Bragança Paulista SP Brazil Universidade São Francisco (USF), Bragança Paulista, SP – Brazil

**Keywords:** Diagnostic Imaging, Biopsy, Genetics, Cardiomyopathies

Increased ventricular wall thickness is a common finding in cardiovascular practice and is frequently attributed either to secondary myocardial hypertrophy or to sarcomeric hypertrophic cardiomyopathy (HCM). However, this phenotype may conceal a broad spectrum of genetic, infiltrative, and storage diseases, each associated with distinct prognostic implications and therapeutic strategies. Accurate differentiation between true myocardial hypertrophy and HCM phenocopies—or genocopies—remains a major diagnostic challenge and requires a multimodality approach integrating advanced cardiac imaging, endomyocardial biopsy (EMB), and genetic testing.^1-3^

Cardiac imaging has evolved beyond morphological assessment. Cardiac magnetic resonance (CMR) with tissue characterization has become a cornerstone of noninvasive evaluation in patients with increased myocardial thickness. Parametric mapping techniques, including T1 and T2 mapping and extracellular volume (ECV) quantification, allow the detection of myocardial compositional changes such as edema, fibrosis, and infiltration, providing important diagnostic clues for conditions such as cardiac amyloidosis, Fabry disease, and glycogen storage cardiomyopathies (e.g., PRKAG2-related disease).^4-6^ In addition, specific late gadolinium enhancement patterns may further support the suspicion of non-sarcomeric etiologies.^3,7^

Despite substantial advances in imaging, histopathological assessment obtained through EMB remains the diagnostic gold standard in selected cases ^8^. Tissue analysis enables definitive identification of abnormal deposits, such as amyloid fibrils, as well as ultrastructural abnormalities, including glycogen-filled vacuoles in PRKAG2 cardiomyopathy, which may not be reliably distinguished by imaging alone. EMB is particularly valuable when imaging findings are inconclusive or when confirmation of a specific diagnosis is required to guide disease-modifying therapies.^8,9^

Genetic testing has transformed the diagnostic landscape of inherited cardiomyopathies, enabling confirmation of sarcomeric HCM and identification of its phenocopies.^10-12^ Nevertheless, genetic evaluation introduces additional complexity, including phenotypic overlaps and the frequent detection of variants in multiple genes. In such scenarios, EMB regains critical importance, as histopathological findings may help establish genotype–phenotype correlation and identify the causal variant, thereby informing clinical management and family screening strategies.

In conclusion, the evaluation of increased myocardial wall thickness demands an integrated diagnostic framework.^13^ CMR plays a central role in screening and tissue characterization; however, EMB remains far from obsolete, providing definitive tissue-level diagnosis. When combined with genetic testing, EMB strengthens diagnostic accuracy, clarifies the underlying etiology of apparent "hypertrophy," and supports a precision medicine approach in contemporary cardiomyopathy care.
